# *Psoralea corylifolia* L. Seed Extract Attenuates Nonalcoholic Fatty Liver Disease in High-Fat Diet-Induced Obese Mice

**DOI:** 10.3390/nu8020083

**Published:** 2016-02-06

**Authors:** Eunhui Seo, Yoon Sin Oh, Hee-Sook Jun

**Affiliations:** 1 College of Pharmacy and Gachon Institute of Pharmaceutical Science, Gachon University, Incheon, 21936, Republic of Korea; eunhuiseo@gachon.ac.kr; 2 Lee Gil Ya Cancer and Diabetes Institute, Gachon University, Incheon 21999, Korea; with62@gachon.ac.kr; 3 Gachon Medical Research Institute, Gil Hospital, Incheon 21565, Korea

**Keywords:** diabetes, obesity, nonalcoholic fatty liver disease, *Psoralea corylifolia* L. seed

## Abstract

Nonalcoholic fatty liver disease (NAFLD), along with obesity, is increasing world-wide and is one of the major causes of chronic hepatic disease. The present study evaluated the ameliorative effect of extract of *Psoralea corylifolia* L. seed (PCS) on high fat diet-induced NAFLD in C57BL/6 mice after daily administration at 300 or 500 mg/kg for 12 weeks. Treatment with PCS extract significantly reduced body weight and blood glucose levels and improved glucose tolerance and insulin sensitivity. In addition, PCS extract treatment significantly attenuated lipid accumulation in liver and adipose tissue and reduced serum lipid and hepatic triglyceride levels. Furthermore, the expression of lipogenic genes and inflammatory genes were reduced, and the expression of fat oxidation-related genes was increased in the liver of PCS extract-treated mice compared with control mice. Our study suggests the therapeutic potential of PCS extract for NAFLD by inhibiting lipid accumulation and inflammation in liver.

## 1. Introduction

Metabolic disorders such as obesity and type 2 diabetes have increased worldwide [1]. More than 200 million people suffer from diabetes and more than 1 billion people are overweight or obese [2]. Nonalcoholic fatty liver disease (NAFLD) refers to the spectrum of pathological conditions characterized by fatty infiltration of the liver, ranging from simple lipid accumulation to nonalcoholic steatohepatitis and to the fibrosis and cirrhosis that occur in the absence of alcohol consumption, viral infection, or other specific etiologies [3]. Because of its strong association with obesity and type 2 diabetes mellitus, NAFLD is now widely considered as characteristic of the metabolic syndrome with insulin resistance. A high-fat diet (HFD) is known to be linked to NAFLD, and HFD-induced lipotoxicity induces hepatic insulin resistance, which plays a major role in the pathogenesis of type 2 diabetes [4,5]. Thus, there is a critical need to clarify the mechanisms that mediate the development and progression of NAFLD and type 2 diabetes, and to identify potential therapies for the disease.

Despite of prevalence of NAFLD, effective therapy for NAFLD is not fully established. The current therapy for NAFLD consists of managing body weight, oxidative stress, insulin resistance, and lipid profile. Insulin receptor sensitizing agents such as thiazolidinediones and antioxidants such as Vitamin E have been tested for the treatment of NAFLD [6]. Because NAFLD is associated with several metabolic disorders, targeting drug development for NAFLD may be effective against obesity, type 2 diabetes, dyslipidemia, and oxidative stress as well as for the improvement of NAFLD.

Numerous studies have suggested that natural components in plants, especially polyphenols and flavonoids, have insulin sensitizing effects as well as hypolipidemic and antioxidant potential [7]. Thus, natural plant extracts might have multiple modes of action in glucose and lipid metabolism [8]. The seeds of *Psoralea corylifolia* (PCS), commonly known as “Boh-Gol-Zhee” in Korea, have been used in herbal and traditional medicine. Six compounds—bakuchiol, psoralen, isopsoralen, corylifolin, corylin, and psoralidin—are the major components of PCS extract and are potent antioxidants [9]. PCS have been reported to have a multitude of health benefits such as antioxidant [9], anti-hepatotoxic [10,11], anti-tumor [12] and anti-bacterial effects [13,14]. In particular, bakuchiol, a polyphenol compound in PCS, has protective effects against hepatic injury [10,11]. As well, psoralen and isopsoralen, coumarins of PCS, have anti-tumor effects [15] and alleviate amnesia [16]. Furthermore, we found that PCS extracts showed protective effects against oxidative stress-induced pancreatic beta cell apoptosis [17] and hepatic damage [18]. These data and reports suggest that PCS extracts might have ameliorative effects on NAFLD. In this study, we examined whether PCS extracts have beneficial effects against HFD-induced NAFLD in mice.

## 2. Materials and Methods

### 2.1. Preparation of PCS Extract

The PCS used in the present study were purchased from an oriental drug store (Kwang Myung Dang Co., Ulsan, Korea), and the extract was prepared by the standard procedure as described previously [17,18]. In brief, the dried seeds (300 g) were ground into small pieces, and then extracted twice with distilled water under reflux. The combined water extract was evaporated in vacuo to give a dark brownish residue (61.92 g). The same batch of PCS extract was used throughout this study.

### 2.2. Animals

Six-week-old male C57BL/6 mice were supplied by the Orient Bio Inc. (Kyungki-Do, Korea). Animals were maintained at animal facilities at the Lee Gil Ya Cancer and Diabetes Institute, Gachon University of Medicine and Science, under a 12-h light, 12-h dark photoperiod. All animal experiments were carried out under a protocol approved by the Institutional Animal Care and Use Committee at Lee Gil Ya Cancer and Diabetes Institute, Gachon University. After adaptation for one week, mice were provided with either a HFD (60% fat) or regular chow diet (5.4% fat). HFD-fed mice were treated orally with PCS extract (300 or 500 mg/kg/day) or vehicle (water) by gavaging at the same as the HFD feeding began, and treatments continued daily for 12 weeks (*n* = 8–10 in each group).

### 2.3. Measurement of Blood Glucose Levels

After 12 weeks of PCS extract treatment, mice were not fed for 4 h, and then glucose levels were measured in the tail vein blood with a glucose analyzer (OneTouch® Ultra, Lifescan, Johnson & Johnson, Milpitas, CA, USA).

### 2.4. Glucose Tolerance Tests

Animals were fasted overnight and glucose (2 g/kg) was administered by intraperitoneal injection. Blood samples were obtained from the tail vein at 0, 30, 60, 90, 120, 150 and 180 min after glucose loading. Blood glucose levels were measured with a glucose analyzer (OneTouch® Ultra, Lifescan, Johnson & Johnson).

### 2.5. Insulin Tolerance Tests

For insulin tolerance tests, animals were fasted for 4 h and insulin (1 U/kg, Humilin, Lilly, Indianapolis, IN, USA) was administered by intraperitoneal injection. Blood glucose levels were measured at 0, 30, 60 and 90 min after insulin load.

### 2.6. Measurement of Hemoglobin A1c (HbA1c) Levels

HbA1c measurements were made using an AU 680 chemistry analyzer (Beckman Coulter, Inc. Brea, CA, USA) and an HbA1c APT kit (Beckman Coulter, Inc.) following the manufacturer’s instructions. HbA1c < 6% is considered normal [19]. 

### 2.7. Serum Lipid Profile

After 12 weeks of PCS extract treatment, blood samples were collected after 4 h of food deprivation. Blood samples were centrifuged at 3000 *g* for 20 min, and serum was collected. Serum levels of total cholesterol, triglycerides, low-density lipoprotein (LDL)-cholesterol and high-density lipoprotein (HDL)-cholesterol were measured using Beckman Coulter AU680 chemistry analyzer (Beckman Coulter, Inc. Brea, CA, USA).

### 2.8. Oil Red O Staining

Liver pieces were embedded in optimal cutting temperature compound. Frozen liver sections were cut at 10 μm thickness, fixed with 10% buffered formalin, dehydrated with 100% propylene glycol, and stained with 0.5% Oil Red O for 30 min at 55 °C. Sections were washed repeatedly with 85% propylene glycol, followed by distilled water. Then, sections were stained with hematoxylin. Lipid droplets were stained red.

### 2.9. Hematoxylin and Eosin Staining

The adipose tissues were fixed in 10% neutral buffered formalin, embedded in paraffin, and stained with hematoxylin and eosin. For staining, slides were deparaffinized by incubation in xylene, hydrated in a series of ethanol (100%, 95%, 80%, and 70%), washed in distilled water and stained with hematoxylin (Sigma-Aldrich, St. Louis, MO, USA) and followed by eosin (Sigma-Aldrich). After washing, sections were rapidly dehydrated in an ethanol series. Finally, the sections were washed in xylene and mounted. 

### 2.10. Quantification of Liver Triglyceride Content

Liver tissue (50 mg) was digested with ethanolic KOH (2 parts EtOH: 1 part 30% KOH) overnight, and then KOH and distilled water were added to the digested solution. After centrifugation (1000 *g* for 5 min), the supernatant was transferred into a new microtube and mixed with 1 M MgCl_2_. The sample was incubated for 10 min on ice and then centrifuged at 1000 *g* for 5 min. Triglyceride content was measured in the upper phase solution using a TG-S kit (Asan Pharmaceutical Company, Seoul, Korea).

### 2.11. Quantitative Real-Time RT-PCR (qRT-PCR) Analysis

The total RNA was extracted from the liver tissue using TRIZOL reagent (Invitrogen Corp., Carlsbad, CA, USA), following the manufacturer’s instructions, and cDNA was synthesized using a PrimeScript 1st strand cDNA synthesis kit (Takara Bio Inc., Kyoto, Japan). qRT-PCR was performed using the SYBR Premix Ex Taq II, ROX plus (Takara Bio Inc.) and the Prism 7900HT sequence detection system (Applied Biosystems, Foster City, CA, USA). PCR was carried out for 40 cycles (2 min at 50 °C, 10 min at 95 °C, and 40 cycles of 10 s at 95 °C and 1 minute at 60 °C). The primer sequences used are shown in Table 1. The relative copy number was calculated using the threshold crossing point (*C*t) as calculated by ΔΔCt.

**Table 1:** Primers used for quantitative real-time PCR.

Gene	Forward/Reverse Primers
*cyclophilin*	5′- TGGAGAGCACCAAGACAGACA 5′-TGCCGGAGTCGACAATGAT
*SREBP1c*	5′-TGCCATCGCCAAGGAGTAG 5′-GGCCCGGGAAGTCACTGT
*SCD1*	5′-CCGGAGACCCTTAGATCGA 5′-GCCTGTAAAAGATTTCTGCAAACC
*FAS*	5′-GCTGCGGAAACTTCAGGAAAT 5′-AGAGACGTGTCACTCCTGGACTT
*CPT1α*	5′-CAAAGATCAATCGGACCCTAGAC 5′-CGCCACTCACGATGTTCTTC
*PGC1α*	5′-CACTGACAGATGGAGCCGTGA 5′-TGTTGGCTGGTGCCAGTAAGAG
*IL1β*	5′-CTACAGGCTCCGAGATGAACAAC 5′-TCCATTGAGGTGGAGAGCTTTC
*MCP1*	5′-TTAAAAACCTGGATCGGAACCAA 5′-GCATTAGCTTCAGATTTACGGG
*SOCS3*	5′-TCCAGCATCTTTGTCGGAAGA 5′-CCAGGCAGCTGGGTCACTT

### 2.12. Western Blotting

Cells were solubilized with Mammalian Protein Extraction Buffer (GE Healthcare, Milwaukee, WI, USA) containing a protease and phosphatase inhibitor cocktail (Sigma-Aldrich). Proteins (30–50 μg) were resolved by 8% or 15% sodium dodecyl sulfate polyacrylamide gel electrophoresis, transferred onto membranes, and blocked with tris buffered saline containing Tween 20 in 5% non-fat dry milk. The membranes were incubated with specific primary antibodies and visualized by incubating with horseradish peroxidase-conjugated secondary antibodies (Santa Cruz Biotechnology Inc., Santa Cruz, CA, USA). Antibodies against *carnitine palmitoyltransferase* *(CPT)* *1* were obtained from Santa Cruz Biotechnology Inc. (Santa Cruz Biotechnology Inc.). Antibodies against *β-actin* and *peroxisome proliferator-activated receptor* *γ coactivator* *(PGC) 1α* were obtained from Sigma-Aldrich (St. Louis, MO, USA) and Abcam (Cambridge, MA, USA), respectively. Chemiluminescence was detected by LAS-4000 (Fuji Film, Tokyo, Japan) after adding Immobilon Western Chemiluminescent HRP Substrate (Millipore, St. Charles, MO, USA).

### 2.13. Statistical Analyses

All data are expressed as mean ± standard error of at least three independent experiments. Data were analyzed using Analysis of Variance followed by *post-hoc* analysis using the Tukey range test (SPSS 10.0 statistical software, SPSS Inc., Chicago, IL, USA). *p*-values less than 0.05 were considered statistically significant.

## 3. Results

### 3.1. PCS Extract Treatment Decreased Hemoglobin A1c, Blood Glucose Levels, and Body Weight Gain in HFD-Fed Mice

**Figure 1:**
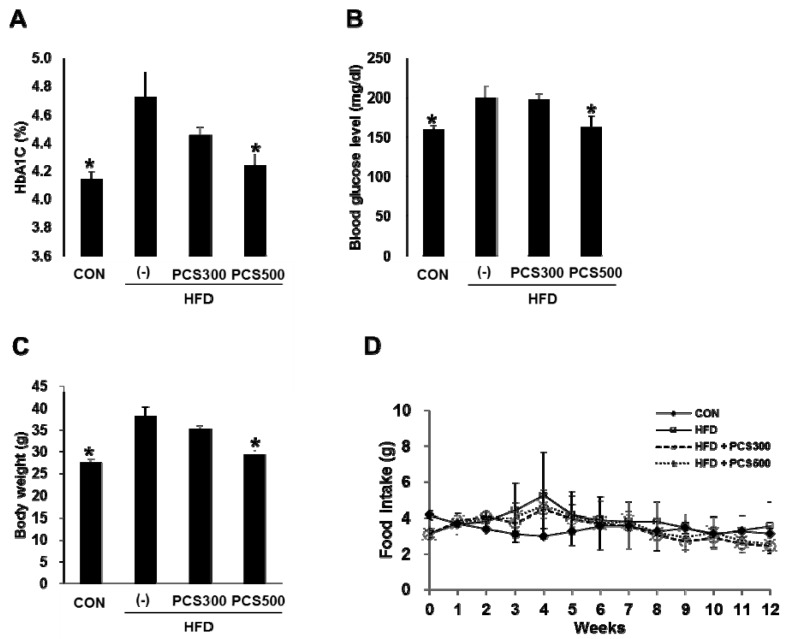
Effect of *Psoralea corylifolia* L. seed (PCS) extract on HbA1c, blood glucose and body weight gain in HFD-fed mice. Mice were fed with regular chow diet (CON) or HFD for 12 weeks. HFD-fed were treated with vehicle (‒) or PCS extract (300 or 500 mg/kg/day) from the first day of HFD feeding (*n* = 7–10/group). (**A**) HbA1c levels; (**B**) Blood glucose levels; and (**C**) body weight after 12 weeks of PCS extract treatment; (**D**) Food intake during 12 weeks of PCS extract treatment. *, *p* < 0.05 *vs.* vehicle-treated HFD-fed mice.

PCS extract (300 mg/kg/day or 500 mg/kg/day) was administered into C57BL/6 mice for 12 weeks during HFD feeding, and we compared changes in HbA1c levels, blood glucose levels, and body weight. The mice fed with HFD showed significantly increased HbA1c (Figure 1A), blood glucose levels (Figure 1B) and body weights (Figure 1C) compared with regular chow diet-fed control mice. Treatment with PCS extract in HFD-fed mice reduced HbA1c and blood glucose levels, and body weight gain dose dependently with a significant reduction at a dose of 500 mg/kg/day compared with vehicle-treated HFD-fed mice (Fig 1 A–C). There were no differences in food intake among the vehicle and PSC extract treatment groups in HFD-fed mice (Figure 1D).

### 3.2. PCS Extract Treatment Improved Insulin Sensitivity and Glucose Tolerance in HFD-Fed Mice

**Figure 2:**
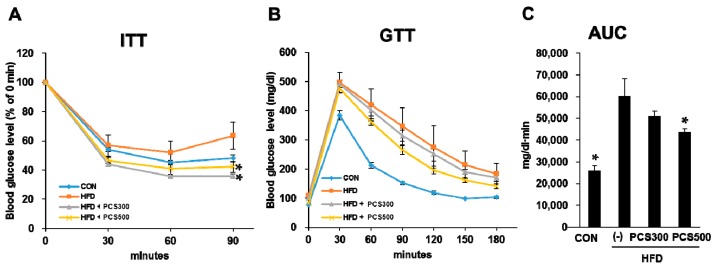
Effect of PCS extract on insulin tolerance and glucose tolerance in HFD-fed mice. Mice were fed with regular chow diet (CON) or HFD for 12 weeks. HFD-fed were treated with vehicle or PCS extract (300 or 500 mg/kg/day) from the first day of HFD feeding (*n* = 7–10/group). (**A**) Insulin tolerance tests (ITT) were performed after 12 weeks of PCS treatment. Blood glucose levels were measured at the indicated times after insulin injection (1 U/kg Intraperitoneal (i.p.)); (**B**) Glucose tolerance tests (GTT). Blood glucose levels were measured at the indicated times after glucose load (2 g/kg i.p.); (**C**) Area under the curve (AUC) of GTT graph. * *p* < 0.05 *vs.* vehicle-treated HFD-fed mice.

To address whether PCS extract treatment improved insulin sensitivity, we performed insulin tolerance tests after 12 weeks of PCS treatment. PCS extract-treated HFD-fed mice showed an enhanced reduction in glucose levels in response to exogenous insulin at 90 min following insulin injection compared with vehicle-treated HFD-fed mice, indicating that insulin sensitivity was improved by PCS extract treatment (Figure 2A). To determine whether blood glucose levels are properly controlled in PCS extract-treated mice, we performed glucose tolerance tests after 12 weeks of PCS treatment. Intraperitoneal glucose tolerance tests showed that a glucose load given to the normal control group produced a rapid increase in blood glucose levels at 30 min which returned to baseline values within 120 min (Figure 2B). All HFD-fed mice showed hyperglycemia above 400 mg/dL 30 min after glucose loading. However, treatment with PCS extracts improved glucose tolerance dose dependently and treatment with 500 mg/kg/day of PCS extract resulted in a significant improvement in glucose tolerance compared with the vehicle-treated HFD-fed mice (Figure 2C).

### 3.3. PCS Extract Treatment Decreased Plasma Lipid Profiles in HFD-Fed Mice

**Figure 3:**
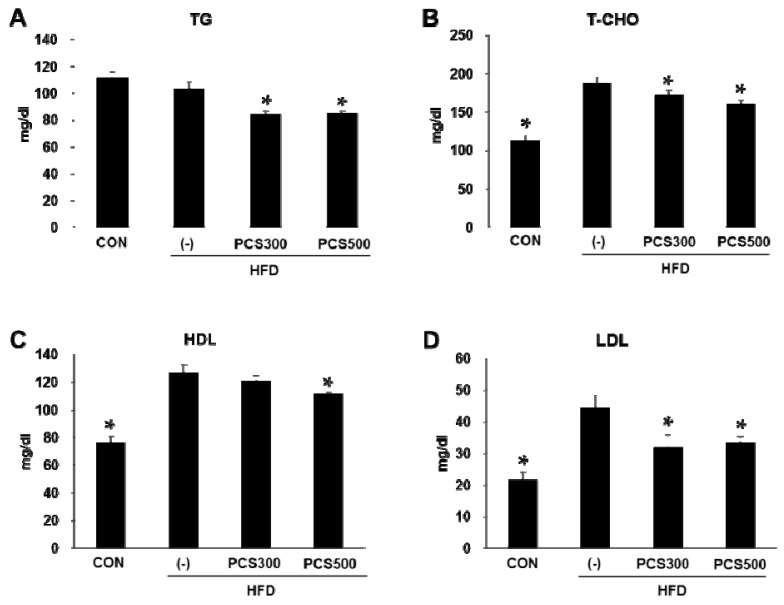
Effect of PCS extract on serum TG and cholesterol levels. Mice were fed with regular chow diet (CON) or HFD for 12 weeks. HFD-fed were treated with vehicle (−) or PCS extract (300 or 500 mg/kg/day) from the first day of HFD feeding (*n* = 7–10/group). (**A**) Triglyceride levels (TG); (**B**) Total cholesterol levels (T-CHO); (**C**) HDL-cholesterol levels; and (**D**) LDL-cholesterol levels in sera were measured after 12 weeks after PCS extract treatment. * *p* < 0.05 *vs.* vehicle-treated HFD-fed mice.

We next investigated whether there are any changes in serum lipid levels after PCS extract treatment. Serum triglyceride levels were not increased by HFD, but PCS extract treatment significantly decreased serum triglyceride levels at a dose of 300 or 500 mg/kg/day (Figure 3A). Total cholesterol (Figure 3B), HDL-cholesterol (Figure 3C), and LDL-cholesterol (Figure 3D) levels were significantly increased in HFD-fed mice as compared with regular chow diet-fed control mice. PCS extract treatment at both doses (300 and 500 mg/kg/day) significantly inhibited this increase of total cholesterol and LDL-cholesterol. HDL-cholesterol levels were slightly, but significantly inhibited by treatment with 500 mg/kg PCS extract (Figure 3B–D). 

### 3.4. PCS Extract Treatment Decreased Lipid Accumulation in Liver and Adipose Tissue in HFD-Fed Mice

**Figure 4:**
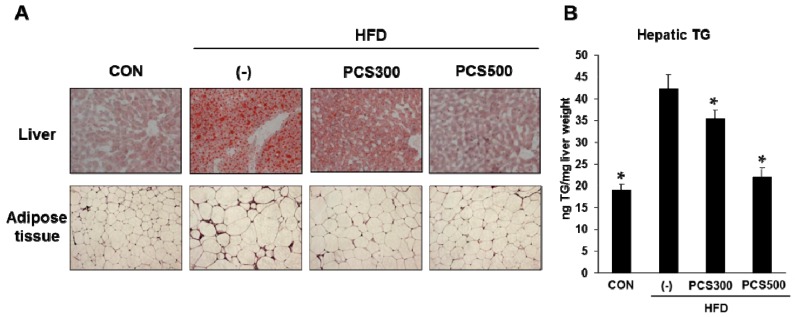
Effect of PCS extract on lipid accumulation in liver and adipose tissues. Mice were fed with regular chow diet (CON) or HFD for 12 weeks. HFD-fed mice were treated with vehicle (−) or PCS extract (300 or 500 mg/kg/day) from the first day of HFD feeding (*n* = 7–10/group). (**A**) Histology of liver and adipose tissue. Liver sections were stained with Oil red-O and adipose tissue sections were stained with hematoxylin and eosin. Magnification 200×; (**B**) Hepatic triglyceride level (TG). * *p* < 0.05 *vs.* vehicle-treated HFD-fed mice.

The accumulation of hepatic lipid during a HFD is a major cause of NAFLD [20]. To investigate the effects of PCS extract on the development of NAFLD in HFD-fed mice, we assessed the lipid content in the liver. Lipid droplet accumulation was obviously increased in HFD-fed mice, whereas the accumulation of lipid droplets was reduced in 300 or 500 mg/kg PCS extract-treated mice compared with vehicle-treated mice (Figure 4A). Hepatic triglyceride levels, which were increased in HFD-fed mice, were significantly lower in PCS extract-treated mice (Figure 4B). Histological analysis of epididymal adipose tissue sections by hematoxylin and eosin staining also showed smaller adipocytes in PCS extract-treated HFD-fed mice than in vehicle-treated HFD-fed mice (Figure 4A).

### 3.5. PCS Extract Treatment Decreased mRNA and Protein Expression of Genes for Lipid Metabolism and Hepatic Inflammation in Liver of HFD-Fed Mice

**Figure 5:**
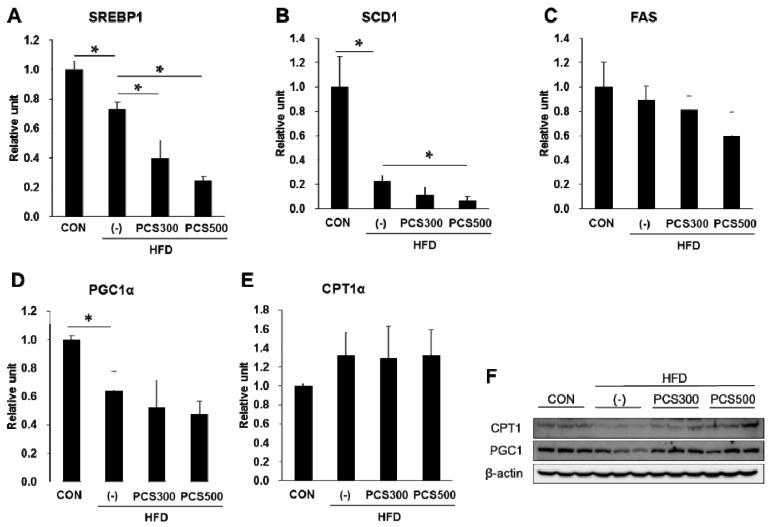
Effect of PCS extract on mRNA and protein expression of lipid metabolism in liver. Mice were fed with regular chow diet (CON) or HFD for 12 weeks. HFD-fed mice were treated with vehicle (−) or PCS extract (300 or 500 mg/kg/day) from the first day of HFD feeding (*n* = 7–10/group). After 12 weeks of PCS extract treatment, total RNA was extracted from the liver tissue and qRT-PCR analysis was performed for (**A**) *sterol regulatory element binding protein (SREBP1)*; (**B**) *stearoyl-coenzyme A desaturase (SCD1)*; (**C**) *fatty acid synthase (FAS)*; (**D**) *proliferator-activated receptor γ coactivator (PGC1α)*; and (**E**) *carnitine palmitoyltransferase* *(CPT1α)*; (**F**) Total protein was prepared and Western blotting analysis was carried out for *CPT1* and *PGC1*. * *p* < 0.05 *vs.* vehicle-treated HFD-fed mice.

To investigate *de novo* lipogenesis in PCS extract treated mice, we measured the expression of *sterol regulatory element binding protein (SREBP)-1c*, *stearoyl-coenzyme A desaturase (SCD) 1* and *fatty acid synthase (FAS)* mRNA, which are involved in lipogenesis. We found that *SREBP1c* and *SCD1* mRNA levels were significantly decreased in PCS extract-treated HFD-fed mice as compared with vehicle-treated HFD-fed mice (Figure 5A,B). *FAS* mRNA levels declined in PCS extract-treated HFD-fed mice but this was not significant (Figure 5C). 

A previous *in vitro* study showed that PCS extract could induce activation of mitochondrial function and synthesis [18]. Therefore, we examined the effects of PCS extracts on the mRNA and protein expression of *PGC1α* and *CPT1α*, which are involved in fatty acid oxidation, in the liver of PCS extract-treated mice. mRNA expression of *PGC1α* (Figure 5D) and *CPT1α* (Figure 5E) was not changed by PCS extract treatment, but protein levels of *PGC1α* and *CPT1α* were increased in the liver of PCS-treated mice compared with vehicle-treated mice (Figure 5F).

**Figure 6:**
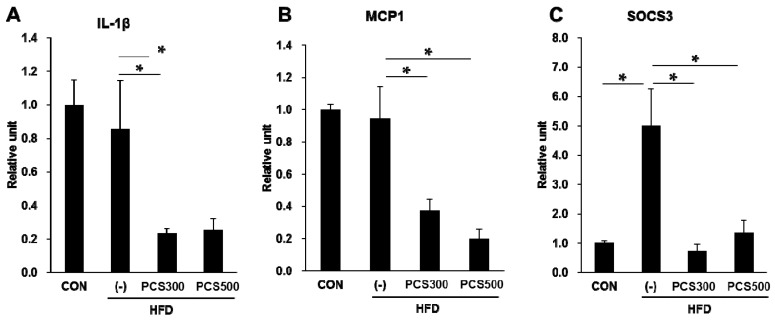
Effect of PCS extract on mRNA and protein expression of hepatic inflammation. Mice were fed with regular chow diet (CON) or HFD for 12 weeks. HFD-fed were treated with vehicle (−) or PCS extract (300 or 500 mg/kg/day) from the first day of HFD feeding (*n* = 7–10/group). After 12 weeks of PCS extract treatment, total RNA was extracted from the liver tissue qRT-PCR analysis was performed for (**A**) *interleukin-1β (IL-1β)*; (**B**) *monocyte chemoattractant protein 1 (MCP1)*; and (**C**) *suppressor of cytokine signaling 3 (SOCS3)*. * *p* < 0.05 *vs.* vehicle-treated HFD-fed mice.

The proinflammatory cytokines are associated with the pathogenesis of NAFLD and contribute to the increased risk for nonalcoholic steatohepatitis and liver cirrhosis [21]. To investigate whether PCS extract treatment affects liver inflammation, we measured the expression of inflammatory molecules such as *interleukin (IL)-1β*, *monocyte chemoattractant protein (MCP) 1* and *suppressor of cytokine signaling (SOCS) 3*. We found that mRNA expression of *IL-1β* (Figure 6A), *MCP1* (Figure 6B) and *SOCS3* (Figure 6C) was significantly decreased in PCS extract-treated HFD-fed mice as compared with vehicle-treated HFD-fed mice.

## 4. Discussion

Excessive energy consumption induces fat accumulation in adipose tissue and the liver, leading to obesity and fatty liver disease [22], which are emerging health problems. Natural products including green tea extract, coffee and several extracts of medical plants have been hypothesized to prevent NAFLD or its progression via several mechanisms, such as sensitizing insulin effects, activating adiponectin expression, and down-regulating pro-inflammatory cytokines, by antioxidant effects or by anti-dyslipidemic properties [23,24,25].

The seeds of *Psoralea corylifolia* (PCS), commonly known as “Boh-Gol-Zhee” in Korea, have been used in herbal and traditional medicine. In a previous study, we found that PCS extract has anti-oxidative effects in hepatocytes and pancreatic β-cells [17,18]. NAFLD patients’ lowered antioxidant capacity has led to the idea that PCS extract might have beneficial effects on NAFLD.

According to the “multihit” hypothesis, disrupted lipid metabolism and insulin resistance are the first step towards NAFLD development [26]. In our study, HFD-fed mice showed a significant increase of serum lipid and blood glucose levels, impaired glucose tolerance and insulin resistance. However, hyperglycemia, increase of HbA1c levels, glucose intolerance and insulin resistance associated with HFD were ameliorated by PCS extract treatment. High levels of serum lipid mediates lipotoxicity by inducing lipid over-accumulation through insulin resistance [27]. In our study, PCS extract treatment significantly lowered the HFD-induced rise in serum lipid levels, indicating that the decrease of serum lipid might have contributed to ameliorating insulin resistance, resulting in lowered free fatty acids (FFA) influx to liver.

The liver plays an important role in whole-body energy homeostasis, and thus, its functional disorder has relevance for metabolic syndrome and diabetes. The liver not only takes up FFA from the diet and adipose tissue, but also participates in the *de novo* synthesis of FFA by their conversion into triglycerides through esterification. Also, hepatic triglycerides can be released again into circulation as very low-density lipoproteins, and excess FFA in liver which are not synthesized into triglycerides are used by β-oxidation [28]. NAFLD occurs when this regulation is disrupted in the liver, leading to hepatic steatosis [29]. *SREBP1c*, a key player in hepatic lipogenesis, activates nearly all genes required for *de novo* synthesis of fatty acid and triglyceride synthesis [30], and *SCD1* is the rate-limiting enzyme involved in the biosynthesis of monounsaturated fatty acids [31]. Unexpectedly, HFD significantly decreased the gene expression of *SREBP1c* and *SCD1* compared with regular chow diet, possibly because excessive fat was already present and there was no need for lipid synthesis. In fact, similar results have been reported [32,33] and it was reported that HFD did not induce lipogenic gene expression, despite fatty liver induction [34,35]. These differences in lipogenic gene expression might be dependent on the duration of HFD. Regardless, the expression of *SREBP1c* and *SCD1* mRNA was further decreased by PCS extract treatment.

*CPT1* is associated with the mitochondrial outer membrane and regulates energy production from the main oxidative substrates [36]. *PGC1α* controls many aspects of lipid β-oxidation, mitochondrial biogenesis and respiration [37]. Protein levels of *CPT1* and *PGC1α* were increased by PCS extract treatment. These results suggest that PCS extract treatment improves NAFLD through regulation of overall hepatic lipid metabolism.

It is known that PCS extract and bakuchiol, which is the main component of PCS, have anti-inflammatory effect in macrophages [38]. Hepatic inflammation is a critical event in the progression of NAFLD and may exacerbate lipid-mediated injuries [39]. Because NAFLD is strongly associated with hepatic inflammation, the gene expression of inflammatory markers were measured in the liver of PCS extract-treated mice given a HFD. As in the case of lipogenic gene expression, the HFD did not induce the mRNA expression of *IL-1β* and *MCP1* in the present study, which agrees with the study of Lei Zhao *et al.* [40]. It is possible that a HFD was not enough to induce nonalcoholic steatohepatitis and hepatic inflammation in the fatty liver. However, a HFD did induce the expression of *SOCS3*. PCS treatment reduced the gene expression of *IL-1β*, *MCP1* and *SOCS3*. 

## 5. Conclusions

In conclusion, we showed the ameliorative effects of PCS extract on HFD-induced NAFLD in mice. PCS extract treatment decreased body weight gain and serum lipid levels in HFD-fed mice. Lipid accumulation in liver and adipose tissue was decreased probably due to the decrease of lipogenic gene expression and increase of lipid β-oxidation related gene expression. In addition, PCS extract treatment reduced inflammatory gene expression. Thus, these results provide insights into the therapeutic potential of PCS extract in the management of NAFLD.

## Abbreviations

CPT1α: carnitine palmitoyltransferase 1; FAS: fatty acid synthase; FFA: free fatty acids; HbA1c: hemoglobin A1c; HDL: high density lipoprotein; HFD: high fat diet; IL1β: interleukin 1β; LDL: low density lipoprotein; MCP1: monocyte chemoattractant protein; NAFLD: nonalcoholic fatty liver disease; PCS: *Psoralea corylifolia* L. seed; PGC1α: peroxisome proliferator-activated receptor γ coactivator 1α; ROS: reactive oxygen species; SCD1: stearoyl-coenzyme A desaturase 1; SOCS3: suppressor of cytokine signaling; SREBP1: sterol regulatory element binding protein 1.
